# Population network structures, graph theory, algorithms to match subgraphs may lead to better clustering of households and communities in epidemiological studies

**DOI:** 10.1017/S0950268819002255

**Published:** 2020-01-21

**Authors:** Arni S. R. Srinivasa Rao

**Affiliations:** Division of Health Economics and Modeling, Department of Population Health Sciences, Director – Laboratory for Theory and Mathematical Modeling, Department of Medicine – Division of Infectious Diseases, Medical College of Georgia, Department of Mathematics, Augusta University, 1120, 15th Street, AE 1015, Augusta, GA 30912, USA

Sir,

One of the important preliminary tasks for formulating epidemic control programme interventions is the identifications of clusters that drive an epidemic in a population. Information about seroprevalence within various groups in the population is often one of the critical requirements for clustering in epidemiological studies. The article by Trickey *et al*. [1] (on 7 October 2019) applied regression-based approaches and simulations to investigate the clusters of household communities for the presence of hepatitis C virus antibodies primarily from the data available through a population-based seroprevalence survey [2]. I appreciate the article [1] and the methods they adopted in investigating the clusters. I also agree that more research is needed to understand the reasons why a specific set of clusters has been obtained from amongst a large number of alternatively delineated clusters.

Even if we assume that the survey data [2] and corresponding weights are as accurate as possible (given general limitations applicable to any large-scale population-based surveys and not specific to their paper), the data collected might not be sufficient to optimally identify the formation of the clusters. Different analytical approaches to the present data may also be very profitably employed. Regression-based approaches, allocating certain probability distributions to seropositive individuals and conducting repeated simulations might not be adequate for obtaining better insights into the process of cluster formation. Optimally identifying clusters is essentially a statistical classification problem, and alternative approaches that successfully handle clustering may also be fruitfully employed here. Thus, for example, a well-defined network on injection-sharing individuals can be constructed from serosurvey data that has individual-level social, demographic, clinical information, etc. This can be used to construct a directed or undirected finite graph, say, *G*(*n*,*m*) with *n* number of vertices and *m* number of edges. Each vertex represents an individual in the injection-sharing network, and the number of edges in the network is the individuals that share injections among them. Two vertices corresponding to two individuals are connected by an edge if, and only if, they share an injection; the arrow can also indicate if the sharing is a two-way process or one-way process if the data are available to make this a directed graph. Using algorithms to match the patterns within the finite graph constructed, one could provide useful information on drivers and clusters of the spread of epidemics, such as hepatitis C, HIV, etc. Such information will be indispensable in formulating effective epidemic control programmes and targeted interventions. The network methods developed by us in the course of our analysis of the spread of parasites [3, 4] have proven to help investigate the diversity of parasites as studied in Blake *et al*. [5].

**Fig. 1.**
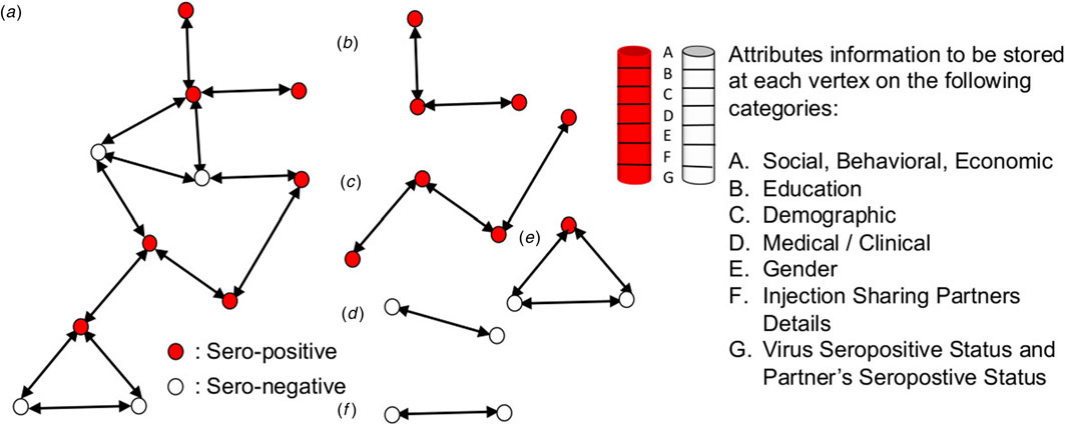
Graphs and subgraphs in a hypothetical injection sharing network. Let (a) be the graph *G* (*n*, *m*) for *n* = 11, *m* = 13. (b) to (f) are example subgraphs constructed from the graph in (a) based on the attributes information at the vertices. Red and hollow cylinders on the right indicate vertices with stored information on attributes A to G.

The originality that we would like to bring into this graph structure is to store attribute-level information at each vertex. Let *G*_*i*_ (*p*_*i*_, *m*_*i*_) be the *i*^th^ subgraph with the *p*_*i*_ number of vertices and *m*_*i*_ number of edges formed from a subset of vertices of *G* (*n*, *m*) for *i* = 1, 2, …, *k* such that 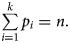 Let the vertex *p*_*i*_ is associated with the information on the presence or absence of pre-determined attributes that were collected during the population-based serosurvey. Figure 1 has the information for a list of categories from which attribute-level information will be collected and stored at the vertex *p*_*i*_. The attribute information stored at each vertex could help provide the qualitative features of selecting a partner or being selected by a partner to share injection. The graph *G* (*n*, *m*) and its set of all subgraphs *G*_*i*_ (*p*_*i*_, *m*_*i*_) for *i* = 1, 2, …, *k* will have complete information on attributes and seroprevalence information that was collected through a serosurvey.

Once the population network structures are formed and vertices of transmission dynamics within this network are identified, then either the complete network structure formed or subgraphs can be used to identify similar structures or subgraphs in populations where network information is available but not the status of seropositivity. This identification is similar to the subgraph isomorphism problem in graph theory, which can be summarised as follows: suppose two graphs *G*_1_ and *G*_2_ are constructed based on two population surveys, then isomorphism of these two graphs means there is a 1-1 correspondence between the vertices of *G*_1_ and *G*_2_. Such identification can be achieved through efficient matching algorithms. One such example can be found in Bonnici *et al*. [6] for matching biochemical networks.

Regression models are powerful statistical tools but they could at most give an average sense of the attributes in Figure 1 that are associated with seropositive or seronegative individuals. For the optimal clustering, such models could be less effective in formulating policies for targeted interventions to prevent new infections in small-sized clusters. The proposed idea of attribute linked finite graphs could help identify optimum clusters within the networks. The author can be reached through an email for further technicalities on the method proposed.
